# Use and misuse of random forest variable importance metrics in medicine: demonstrations through incident stroke prediction

**DOI:** 10.1186/s12874-023-01965-x

**Published:** 2023-06-19

**Authors:** Meredith L. Wallace, Lucas Mentch, Bradley J. Wheeler, Amanda L. Tapia, Marc Richards, Siyu Zhou, Lixia Yi, Susan Redline, Daniel J. Buysse

**Affiliations:** 1 Department of Psychiatry, University of Pittsburgh, 3811 O’Hara Street, Pittsburgh, PA 15231 USA; 2 Department of Statistics, University of Pittsburgh, Pittsburgh, PA USA; 3 School of Computing and Information, University of Pittsburgh, Pittsburgh, PA USA; 4 Brigham and Women’s Hospital, Harvard Medical School, Boston, MA USA

**Keywords:** Feature importance, Polysomnography, Sleep, Random forest, Knockoff variable importance

## Abstract

**Background:**

Machine learning tools such as random forests provide important opportunities for modeling large, complex modern data generated in medicine. Unfortunately, when it comes to understanding *why* machine learning models are predictive, applied research continues to rely on ‘out of bag’ (OOB) variable importance metrics (VIMPs) that are known to have considerable shortcomings within the statistics community. After explaining the limitations of OOB VIMPs – including bias towards correlated features and limited interpretability – we describe a modern approach called ‘knockoff VIMPs’ and explain its advantages.

**Methods:**

We first evaluate current VIMP practices through an in-depth literature review of 50 recent random forest manuscripts. Next, we recommend organized and interpretable strategies for analysis with knockoff VIMPs, including computing them for groups of features and considering multiple model performance metrics. To demonstrate methods, we develop a random forest to predict 5-year incident stroke in the Sleep Heart Health Study and compare results based on OOB and knockoff VIMPs.

**Results:**

Nearly all papers in the literature review contained substantial limitations in their use of VIMPs. In our demonstration, using OOB VIMPs for individual variables suggested two highly correlated lung function variables (forced expiratory volume, forced vital capacity) as the best predictors of incident stroke, followed by age and height. Using an organized analytic approach that considered knockoff VIMPs of both groups of features and individual features, the largest contributions to model sensitivity were medications (especially cardiovascular) and measured medical risk factors, while the largest contributions to model specificity were age, diastolic blood pressure, self-reported medical risk factors, polysomnography features, and pack-years of smoking. Thus, we reach very different conclusions about stroke risk factors using OOB VIMPs versus knockoff VIMPs.

**Conclusions:**

The near-ubiquitous reliance on OOB VIMPs may provide misleading results for researchers who use such methods to guide their research. Given the rapid pace of scientific inquiry using machine learning, it is essential to bring modern knockoff VIMPs that are interpretable and unbiased into widespread applied practice to steer researchers using random forest machine learning toward more meaningful results.

**Supplementary Information:**

The online version contains supplementary material available at 10.1186/s12874-023-01965-x.

## Background

The amount of health data available for analysis has proliferated in recent years, along with the use of machine learning (ML) to model complex associations in high-dimensional data sets. Random forests are among the most accurate ML models, and are continuing to grow in popularity [1]. Since Breiman’s publication of his original random forest manuscript in 2001 [2], it has been cited over 40,000 times in peer-reviewed journals, with approximately 80% of these citations occurring in the last five years alone. But the popularity of random forests – and ML in general – neglect a major drawback: it is difficult to look inside the “black box” to determine which factors are driving the predictive abilities of the algorithm. Such knowledge is exceedingly important for researchers who want to understand not just *whether* a predictive algorithm works, but *why* it works. (See Supplement for details on random forest methods.)

Recognizing the need to understand which features drive the predictive abilities of the random forest, Breiman originally proposed an ad hoc and computationally efficient approach called ‘out of bag’ Variable Importance (OOB VIMP) [2]. OOB VIMPs continue to be the default in most random forest software and are commonly used by applied researchers, despite serious drawbacks that are well-documented in the statistics community [3–6]. Specifically, they have limited interpretability and groups of correlated features tend to have inflated OOB VIMPs. This latter problem is particularly troublesome since complex high-dimensional data nearly always contain correlated features, and it is precisely these settings in which researchers most often turn to random forests.

Alternative VIMPs that address these limitations have been developed in the last few years [7, 8]. However, such methodological discoveries can often take a decade or more to fully integrate in the scientific community. The random forest itself took over 15 years to gain popularity in applied research. The case of the Least Absolute Shrinkage and Selection Operator (LASSO) [9], another popular modeling approach, also exemplifies the problem of slow uptake of methodological discoveries. Nearly 99% of the > 46,000 LASSO citations came more than a decade after the method was first published in 1996. Given the accessibility of large data sets and the ability to use random forests without a full appreciation of their underlying assumptions and limitations, we cannot wait a decade for modern and recommended VIMP approaches to trickle into the applied research community.

The goals of this manuscript are to: (1) demonstrate why the common practices surrounding OOB VIMPs in random forests can lead to vague, potentially misleading, and non-reproducible scientific findings; (2) highlight modern VIMP approaches that address limitations of OOB VIMPs but that have not yet been widely applied in medical research; and (3) demonstrate an organized analytic framework for using modern VIMPs to identify key predictors of incident stroke. We hope that our work will expedite the transfer of critically important knowledge from the statistics community to applied researchers by introducing best practices for examining feature importance.

### Scientific motivation: incident stroke prediction

As a scientifically important motivating example, we examine the role of overnight polysomnography (PSG) features for predicting 5-year incident stroke using data from the Sleep Heart Health Study (SHHS) [10] – a large multi-site cohort of community-dwelling older adults. Overnight PSG can produce hundreds of features related to arousals, oxygen saturation, respiratory events, heart rate, and sleep architecture. Many of these PSG features are used to identify obstructive sleep apnea, an emerging modifiable risk factor for incident stroke especially in men [11–14]. Other PSG features such as sleep architecture (e.g., time in specific sleep stages such as Rapid Eye Movement [REM] or N3 sleep), sleep continuity (e.g., time to fall asleep, minutes awake after first falling asleep) or sleep duration may also be predictive, although they are less widely studied as risk factors for stroke [15, 16].

Despite the potential promise of PSG derived metrics for stroke prediction, the use of PSG as a large-scale screening tool is limited because it is more burdensome and expensive relative to other well-established predictors of stroke that are derived by self-report or information widely available in health records, such as age, hypertension, dyslipidemia, diabetes, cardiovascular disease, anthropometry, and lifestyle behaviors [17–21]. We aim to understand the predictive value of PSG sleep measures in relation to other established clinical and self-report measures from the SHHS, the largest US-based research cohort to examine incident stroke.

Given the high-dimensional nature of data within the SHHS – and the observation that sleep measures are often interconnected to one another and to other stroke risk factors including age, smoking, obesity, and hypertension [16] – random forests present a promising alternative to traditional linear modeling approaches because they allow for complex and empirically-driven combinations of features. However, given our particular interest in understanding the role of PSG-derived sleep features relative to other risk factors, we need to unpack the random forest model to determine which features are driving its predictive abilities.

### OOB VIMP methods

The terminology ‘OOB’ originates from the procedure called ‘bagging’ or ‘Bootstrap Aggregating’ [22]. Bagging is the process of combining results from trees generated across multiple bootstrap samples. (A bootstrap sample is a resample of the original training sample, the same size as the original sample, drawn with replacement.) By construction, each bootstrap sample will, with exceedingly high probability: (a) contain duplicates of individuals from the original sample, and (b) exclude some individuals in the original sample. Bootstrap samples that do not contain data from an individual *i* are considered OOB for that observation. OOB VIMPs leverage OOB samples to enhance computational efficiency.

**Fig. 1:**
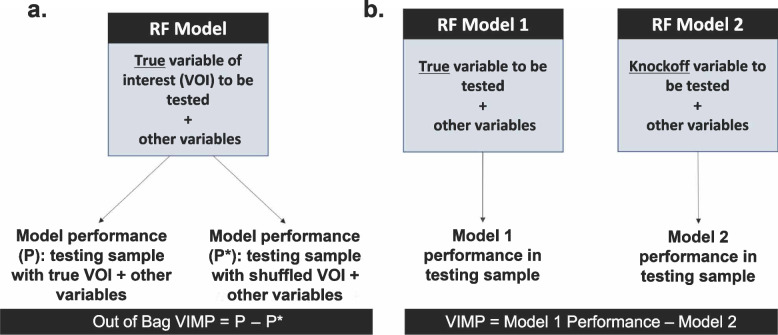
Methods for out of bag (OOB) VIMP (**a**) vs. knockoff VIMP (**b**)

To illustrate how OOB VIMPs are computed, consider a scenario with features X_1_-X_10_ used to predict incident stroke in a sample of individuals *i* = 1,..,N, each with the observation set z_i_ = {x_i1_,…,x_i10_}. The steps to compute the OOB VIMP for X_1_ are as follows (also see Fig. 1a).

1. Construct the random forest in all N participants using X_1_-X_10_ as predictors.
2. For each participant *i*:
  - Use the random forest to predict the outcome for the corresponding observation set z_*i*_ and calculate the accuracy, P_i_.
  - Replace the value of x_i1_ in z_i_ with a randomly selected value of X_1_ while holding x_i2_-x_i10_ at the original values. Denote the new observation set for participant *i* as z_i_* = {x_i1_*, x_i2_,…,x_i10_}.
  - Find all trees in which *i* was not chosen in the resample (i.e., trees grown using the OOB samples for *i*), and thus was not used in the training of that tree. This subset of trees is called the sub-forest for participant *i*.
  - Use the sub-forest for participant *i* to predict the outcome using the observation set z_i_* and denote the accuracy of this prediction by P_i_*. Compute VIMP_i_ = P_i_-P_i_*
3. Compute the VIMP for X_1_ as the average across all VIMP_i_.
4. The VIMPs for the remaining X_2_-X_10_ can be calculated by repeating Step 2–3 and substituting the values of the feature of interest.

The motivation behind the OOB VIMP is straightforward: if X_1_ plays an important role in the model, then randomly changing the value of X_1_ should lead to a change in the prediction and hence a change in the accuracy of that prediction. Conversely, if changing the value of X_1_ has little impact on the predicted value for most observations, then X_1_ would seem to be unimportant.

However, in the years following the introduction of OOB VIMPs, researchers noticed that they can dramatically inflate the importance of features that are highly correlated with other features [3–6]. To explain, consider a simplified example of a random forest used to predict stroke with three features: X_1_, X_2_, and X_3_. Assume that X_1_ and X_2_ are true causal risk factors for stroke and that X_3_ is moderately correlated with X_2_ but not independently related to the outcome. If we grow a random forest using X_1_-X_3_, given the randomness in the forest, X_3_ is likely to be used (split upon) in many trees. Thus, when X_3_ is shuffled to compute the OOB VIMP, there may be a sizeable drop in performance (corresponding to a relatively large OOB VIMP) because X_3_ was used to construct this random forest. However, the OOB VIMP indicates only that X_3_ was used within this specific random forest model. It does not indicate: (a) that X_3_ is needed to predict stroke (it is not needed); (b) whether X_3_ has a relationship with stroke after accounting for other features (it does not); or (c) that if you took out X_3_, the model would perform worse (it may even perform better). In other words, these OOB VIMPs ask about the role of X_3_ in only the model constructed, rather than how well a model *could* have been constructed without relevant information from X_3_.

### Modern VIMP methods

Recent solutions explicitly address the limitations of OOB VIMPs: we broadly denote them as “removal”, “permutation”, and “knockoff” approaches. Removal approaches compare a model with the feature of interest to a model without the feature of interest [8]. Permutation approaches (e.g., Boruta importance) compare the performance of a model with the true feature of interest versus model performance with a permuted feature [7, 23, 24]. Knockoff approaches compare a model with the feature of interest to a model with an altered version of the feature of interest called a knockoff. The knockoff is a simulated version of the variable of interest that has (approximately) the same association with other variables in the model but no true association with the outcome [25], and requires the generation of knockoff variables through a “knockoff generator” [26]. Among the many benefits of these more rigorous modern VIMPs, we note that they can be used with many types of predictive algorithms and can theoretically be computed using any metric of model performance.

The types of VIMPs described above each have different strengths and weaknesses. However, we consider the knockoff approach to be the truest test of a feature’s predictive abilities of a variable of interest within a model because it directly compares the accuracy of a model built using the true feature of interest to the accuracy of a model that is identical in all ways except the feature of interest is not related to the outcome. In particular, the knockoff variables used in the second model may still be correlated with other features that are directly related to the response so long as the feature itself is not directly associated with it. In contrast, when we compare a model with the feature of interest to a model without the feature of interest (i.e., using removal VIMPs), the two models differ in their dependence among variables as well as the number of variables. These alterations can have unexpected consequences, especially when there are dependencies between the feature being tested and the other features in the model. In particular, random forests can sometimes incorporate features that have no relationship to the response whatsoever [27]. For this reason, we focus on knockoff VIMPs for the remainder of this study.

To illustrate how knockoff VIMPs are computed, we again consider our set of features X_1_-X_10_ to predict an outcome. The steps to compute the knockoff VIMP for X_1_ are as follows (also see Fig. 1b).

1. For participants in both training and testing samples, generate a knockoff variable for each feature X_1_-X_10_ using a knockoff generator [25, 26].
2. Construct a random forest model (RF_1_) in a training sample using the true X_1_-X_10_ as predictors.
3. Construct a second random forest model (RF_2_) in the same training sample using the knockoff version of X_1_^*^ and other true predictors X_2_-X_10._
4. For each participant *i* in an independent testing sample:
  - Use RF_1_ to predict the outcome based on their observation set z_i_ = {x_i1_-x_i10_}. Compute the performance *P*_*i1*_.
  - Replace the value of x_i1_ in z_i_ with its knockoff value of X_1_ while holding x_i2_-x_i10_ at the original values. Denote the new observation set as z_i_* = {x_i1_*, x_i2_…,x_i10_}.
  - Use RF_2_ to predict the outcome based on the observation set z_i_*. Compute the performance *P*_*i2*_.
  - Compute VIMP_i_ = P_i1_-P_i2_.
5. Compute the knockoff VIMP for X_1_ as the average across all VIMP_i_.
6. The VIMP for the remaining X_2_-X_10_ can be calculated by repeating Steps 3–5 and substituting the values of the feature of interest.

An essential difference between OOB VIMPs and knockoff VIMPs is that the knockoff VIMP requires two random forest models: one built with the true feature of interest and one built with the altered feature of interest (i.e., the knockoff). In contrast, the OOB VIMP only considers one random forest with the true variable. Thus, while knockoff VIMPs address critical limitations of OOB VIMPs, they require significantly greater computational cost because a new forest must be generated for each knockoff VIMP.

As with all statistical analyses, it is essential to precisely define the scientific question and carefully plan the corresponding analytic strategy when using knockoff VIMPs. Knockoff VIMPs can answer questions of the form: “*How much unique predictive information does the variable (or group of variables) contribute to the ML model, beyond what is offered by the other features in the model?*” Thus, knockoff VIMPs do not determine whether the feature(s) in question *alone* can help predict the response (marginal importance), but rather, can those features *further* improve model performance once the information in the other features are accounted for (conditional importance). For a set of features to be deemed conditionally important, they must have unique predictive information that cannot be explained by complex combinations of any other features in the model. In practice, it can be difficult to show that an individual feature has strong conditional importance, especially when using ML models like random forests that can uncover complex underlying associations. Although knockoff VIMPs are not biased towards interrelated measures, their interpretation still requires careful consideration. If two variables X_1_ and X_2_ are related (even in a complex, non-linear way) and both are important for predicting the outcome, it is possible that their VIMPs will be dampened when examined in the same model simultaneously because one can be used in place of the other for prediction [25].

Advances in modern VIMPs also include formal hypothesis testing procedures to determine whether the calculated VIMPs are statistically different from zero [7, 8]. Thoughtful use of statistical inference for VIMPs may be beneficial when testing pre-specified hypotheses. However, in scenarios that are exploratory and used for hypothesis generation, we recommend emphasizing the magnitude of the VIMP over the p-value, which does not necessarily indicate a meaningful association [28].

## Methods

### Literature review

To evaluate the extent to which applied researchers acknowledge and address the limitations surrounding VIMPs, we performed an in-depth literature review of 50 manuscripts (published between March 2022 and August 2022) that cited the original random forest manuscript [2]. Manuscripts were selected from Web Of Science and were restricted to only English citations and Article or Review Article manuscript types. To focus on health-specific applications, we further restricted our search to manuscripts listed under the following Research Areas: Neurosciences Neurology, Medical Informatics, Radiology Nuclear Medicine Medical Imaging, Pharmacology Pharmacy, Public Environmental Occupational Health, Genetics Heredity, Health Care Sciences Services, Oncology, General Internal Medicine, Psychology, or Psychiatry.

### SHHS demonstration

#### Sample

SHHS participants were recruited from nine existing epidemiological cohort studies focused on cardiovascular risk factors. Several of these cohorts over-sampled individuals who snore, given the study’s focus on sleep-disordered breathing. Those who met the inclusion criteria for SHHS (age 40 years or older; no history of treatment of sleep apnea; no tracheostomy; no current home oxygen therapy) were invited to participate in a baseline examination that included self-report questionnaires, laboratory measurements, and an overnight at-home polysomnogram (PSG). After the initial SHHS visit, participants were followed longitudinally for several health outcomes including adjudicated incident stroke. Parent cohorts provided ongoing surveillance for incident stroke per their specific protocols; additional details on protocols and stroke adjudication is provided elsewhere [14].

The initial SHHS visit included N = 5,804 participants with publicly available data (National Sleep Research Resource; www.sleepdata.org), of whom N = 4,889 (84.2%) had no prior stroke. From the 4,889, we further removed 31 (0.6%) people with > 20% missing data on features of interest, resulting in N = 4,858. With this sample, we used random forest imputation (missForest function and package in R [29]) to impute missing data on features with no more than 20% missing data, considering a total of 641 features measured in SHHS. Our final analytic sample was N = 4,512 participants with no prior stroke and observed follow-up data for the outcome of adjudicated incident stroke in 5 years. Within this final sample, N = 124 (2.7%) participants had incident stroke within 5 years.

#### Variable selection and grouping

Our analytic question is “How much unique information do PSG features contribute to predict incident stroke in the random forest, beyond what is offered by other established clinical and self-report risk factors?” To this end, we studied the literature for established and emerging predictors of stroke and identified 157 features (out of the 641 considered) with potential direct or indirect predictive abilities. We aimed to avoid high levels of redundancy when possible, although we did allow for some highly correlated variables when their combination might have been of interest, and prioritized the selection of features with scientific and/or clinical interpretability. For PSG features – which are of particular interest in our application – we included 15 features that we also considered in a prior manuscript based on their strong clinical and scientific value [24]. Overall, there were relatively low correlations among the pairs of features, with 97.7% of pairs having small spearman correlations < 0.30. However, 44 pairs of features (0.36%) had spearman correlations with magnitudes > 0.70.

Given the relatively high bar of conditional importance, combined with the important considerations for interrelated measures discussed above, we recommend grouping similar features together and testing the conditional importance of the “domain". This approach can enhance the interpretation of findings, amplify VIMP effect sizes, and reduce the competition between individual interrelated variables. When determining which features should be grouped together, we recommend considering one’s scientific questions of interest, which features tap into a similar underlying scientific constructs (regardless of their correlation), and which features may be highly related (linearly or non-linearly) with one another. Knockoff VIMPs do not have a bias towards larger groups of variables, per se. However, if larger groups contain more informative features, we expect the “importance” of that group to be higher because it is more predictive.

Guided by our question of interest, we grouped the 157 features into seven domains based on conceptual, physiological, or methodological similarity (Level 1). We refined these broad domains to create 26 sub-domains of established and emerging stroke risk factors (“Level 2”). Finally, we developed a list of 78 features (or small groups of highly related features) that allowed for a fine-grained examination of specific measures of interest (“Level 3”). Table 1 provides details of the features in the Level 1, Level 2, and Level 3 domains.

**Table 1:** Features included in the random forest. Level 1 domains are in bold. Level 2 domains are indented below Level 1 domains. Level 3 features are italicized, with brackets indicating the number of features if > 1

	Number of Features
**Demographic Characteristics**	**10**
Age	1
Sex	1
Race	3
Other Demographic (*Education, Marital Status [4]*)	5
**Measured Medical Risk Factors**	**28**
Anthropometry (*Body Mass Index, Height, Hip, Neck, Waist, Weight*)	6
Electrocardiogram	15
Blood Pressure (*Systolic, Diastolic*)	2
Lung Function (*Forced Expiratory Volume, Forced Vital Capacity*)	2
Lipids (*HDL, Triglycerides, Total Cholesterol*)	3
**Polysomnography**	**15**
Continuity (*Efficiency, Sleep–Wake Shifts, Wake after Sleep Onset*)	3
Duration (*Sleep Duration, Time in Bed*)	2
REM Sleep (*Percent REM, REM Latency*)	2
Sleep Disordered Breathing (*Apnea–Hypopnea Index, Avg SaO*_*2*_*, Min SaO*_*2*_*, T90*)	4
Sleep Staging (*3/4–1/2 shifts, % Stage 1, % Stage 2, % Stage 3–4*)	4
**Health Behaviors**	**4**
Smoking (*Smoking Status [2], Pack-Years of Smoking*)	3
Alcohol Use	1
**Self-Report Sleep**	**30**
Sleep Apnea Symptoms and Diagnoses (*Ever Snored, Sleep Apnea Diagnoses [2], Stop* *Breathing*)	4
Sleep Health and Disturbances (*Sleep Continuity, Sleepiness [3], Duration [2], Timing [4],* *Insomnia [6], Sleep Disturbances [10]*)	26
**Self-Reported Medical Risk Factors**	**27**
Cardiovascular Disease (*Angina, Coronary Angioplasty, Coronary Artery Bypass Graft, Heart* *Failure, Hypertension, Myocardial Infarction, Pacemaker, Other Heart Surgery*)	8
Diabetes	1
SF-36 Emotional (*Mental Component Total; Mental Health, Role Limitations, Social Functioning* *Subscales*)	4
SF-36 Physical (*Physical Component Total; Body Pain, General Health, Physical Functioning,* *Role Limitations, Vitality Subscales*)	6
Pulmonary Measures (*Asthma [2], Chronic Bronchitis, Chronic Obstructive Pulmonary Disorder,* *Coughing, Emphysema, Oxygen Therapy, Phlegm*)	8
**Medication Use**	**43**
Cardiovascular Medications (*Ace Inhibitors [2], Alpha-Blockers, Anti-Arrhythmics [4],* *Anticoagulants, Beta-Blockers, Calcium Channel Blockers [3], Digitalis, Diuretics [4],* *Nitrates, Nitroglycerine, Phosphodiosterase Inhibitors, Sympathomimetics, Vasodilators [4],* *Anti-hypertensive Medication*)	26
Cholesterol Medications	2
Diabetes Medications	2
Other Medications (*Aspirin [2], Estrogen/Progesterone [3], Gastrointestinal, Non-Steroidal Anti-* *Inflammatory Drugs, Psychiatric [3], Pulmonary [2], Thyroid*)	13

#### Random forest modeling details

We first identified optimal random forest tuning parameters for predicting stroke using SHHS data. For this purpose, we used tenfold cross-validation, ensuring an equal proportion of stroke outcomes in each fold. We trained each random forest (500 trees) as a regression model using the ranger function and package in R [30], with outcomes of stroke assigned a 1 and no stroke assigned a 0. Within each cross-validation fold, we examined all permutations of the “mtry” (number of features available for splitting) and “min.node.size” (minimum node size) parameters to find the values that minimized the root mean squared error (RMSE) of the predictions across the cross-validations. Specifically, we evaluated mtry = m*x for m = 157 predictors and min.node.size = N^y^ for N = 4,512, with x and y each between 0.1 and 1 in steps of 0.1. For our data, x = 0.1 and y = 0.7 were optimal. With these parameters, we also used tenfold cross-validation with Youden’s J index to identify a probability threshold for classifying a prediction as having stroke = 1. The mean (standard deviation) optimal cut-off was 0.033 (0.015). The mean (SD) accuracy, sensitivity, and specificity of the model based on tenfold cross-validation with the optimal parameters were 0.716 (0.012), 0.769 (0.111), and 0.714 (0.013).

#### VIMP computing details

##### OOB VIMPs

We fit a random forest model for categorical stroke using the ranger R function and package [30] and optimal mtry and min.node.size parameters as derived above. We used the “importance = permutation” option to extract OOB VIMPs for each of the 157 features.

##### Knockoff VIMPs

We first generated a knockoff variable for each feature using the create.second_order function in the knockoff package in R to generate multivariate Gaussian knockoff variables [26]. We held out 20% of the sample for testing and retained 80% for training. Using the training data, we grew a random forest with the optimal parameters identified above. For each set of features indicated Table 1, we replaced each variable with its corresponding knockoff and then grew another knockoff random forest with the training data. Using the testing data, we evaluated the sensitivity and specificity of the forests grown using the true versus knockoff variables using the optimal 0.03 threshold, and computed knockoff VIMPs for sensitivity (VIMP_Sens_) and specificity (VIMP_Spec_). Because our own demonstration is exploratory and meant for hypothesis generation, we did not utilize formal statistical inference.

R Version 4.4.2 was used for all analyses.

## Results

### Use of VIMPs in literature

Of the 50 recent publications we reviewed, 43 applied a random forest as the primary analysis, while the remainder cited the random forest manuscript but did not apply the methodology. In 11 of the 43 analyses, authors used data that were clearly publicly available; in ten cases, data availability could not be determined; and in 22 cases, the data were not publicly available (i.e., did not include a URL for data access). In 23 of the 43 manuscripts (53%), authors used random forest VIMPs. Of these 23, eight (35%) failed to mention the type of VIMP used or how it was calculated, 13 (57%) relied at least in part on OOB VIMP measures (though many included an assortment of additional models and measures alongside), one (4%) used split depth, and one (4%) used Boruta importance [23]. Split depth quantifies the number of times a given feature was split on at each depth in the forest, and thus is a similarly problematic measure of feature importance for similar reasons as OOB VIMPs, while Boruta Importance is considered a rigorous modern VIMP approach. Thus, 22 of the 23 manuscripts that incorporated random forest VIMPs utilized an approach known to have serious statistical flaws. Only one used a rigorous approach (Boruta Importance) that improves the quality of interpretation.

Only two of the 50 random forest manuscripts cited any work referencing shortcomings of OOB VIMPs, and two additional works alluded to these kinds of issues without reference. Among these four works that made some mention of potential VIMP-related issues, one attempted to address them directly, two proceeded with OOB VIMPs nonetheless, and one turned to another modeling method entirely. Among the 23 papers employing VIMPs, four attempted to provide statistical inference (confidence intervals and/or hypothesis tests). However, the inference was either applied to an OOB VIMP or the outputs were forced into a classical testing framework (e.g., t-tests) in such a way that it is not clear whether such tests remain statistically valid or even necessarily useful.

### SHHS demonstration

**Fig. 2:**
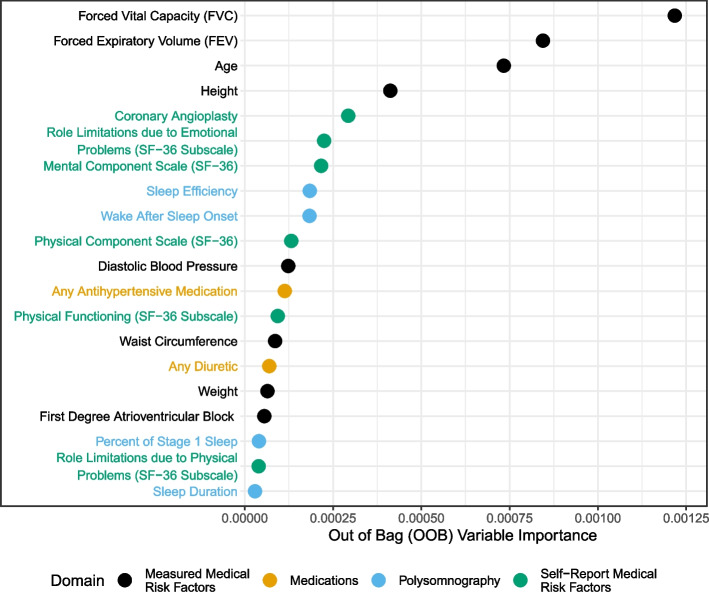
Out of bag (OOB) VIMP results

Figure 2 displays the top 20 OOB VIMPs. Among these, Forced Vital Capacity (FVC) and Forced Expiratory Volume (FEV) (two objective measures of lung function) had the highest VIMPs. Following these were age, height, and history of coronary angioplasty. Other features in the top 20 – with much smaller OOB VIMPs relative to the top five – were: role limitations due to physical limitations (SF-36 subscale), PSG sleep efficiency, PSG wake after sleep onset, and the SF-36 Physical Component scale.

**Fig. 3:**
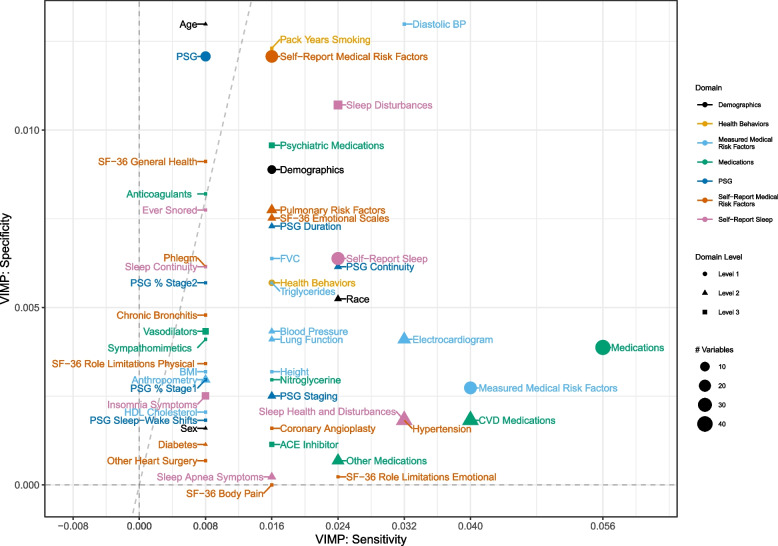
Knockoff VIMP results, showing features (or groups of features) for which the VIMPs based on both sensitivity and specificity are greater than zero

The knockoff VIMPs for Level 1 – Level 3 domains are shown in Fig. 3, which is restricted to domains for which both VIMP_Sens_ and VIMP_Spec_ are above zero to enhance interpretability. Full results for all domains in Table 1 are shown in the Supplement. Overall, the domains generally had a larger contribution to sensitivity (i.e., helped to correctly identify participants with incident stroke) than specificity (i.e., helped to correctly identify participants without incident stroke). Among the broadest Level 1 domains, the 44 medication and 30 measured medical risk factors had the largest contributions to sensitivity (VIMP_Sens_ = 0.056, VIMP_Sens_ = 0.040, respectively) but smaller contributions to specificity relative to other features (VIMP_Spec_ = 0.004, VIMP_Spec_ = 0.003, respectively). The 28 self-report medical risk factors and 15 PSG features had the largest contributions to specificity (VIMP_Spec_ = 0.013 and 0.012, respectively) but smaller contributions to sensitivity relative to other features (VIMP_Sens_ = 0.024 and 0.008, respectively).

Among the more detailed Level 2 and Level 3 domains, age, diastolic blood pressure, and pack years of smoking had the largest contributions to specificity (VIMP_Spec_ = 0.013, 0.013, 0.012 respectively), with roughly similar effect sizes as the Level 1 domains of self-report medical risk factors and PSG. Diastolic blood pressure also had a relatively strong contribution to sensitivity (VIMP_Sens_ = 0.032), and thus stands out as one of the strongest contributors to incident stroke overall. Self-reported sleep disturbances also stood out as having relatively large contributions to both specificity (VIMP_Spec_ = 0.011) and sensitivity VIMP_Sens_ = 0.024).

Regarding PSG features – a primary construct of interest – domains of sleep continuity (efficiency and wake after sleep onset) and sleep duration (duration and time in bed) stood out for their contributions to sensitivity (VIMP_Sens_ = 0.024, 0.016 respectively) and specificity (VIMP_Spec_ = 0.006, 0.007 respectively). REM sleep (percent REM sleep, REM latency) also stood out for its contribution to specificity (VIMP_Spec_ = 0.003).

Considering that the overall average model sensitivity from tenfold cross validation was 0.769, the largest knockoff VIMPs of 0.03 – 0.06 (medications, measured medical risk factors) translate to roughly a 4–7% contribution to the model sensitivity. Other features, including the PSG features of particular interest in this application, altered the model by roughly 0.01–0.02 at most, translating to a 2–3% contribution. While some of these contributions are somewhat small, they are conditional on complex combinations of over 100 other features in the model. Thus, it is still noteworthy that these predictors stand out among the larger set of features.

## Discussion

We provide a review of the limitations surrounding OOB VIMPs in random forests. As an alternative, we provide recommendations for good practice using knockoff VIMPs, which facilitate a direct and interpretable estimate of the value of a feature within a ML model. However, in the context of high-dimensional data, findings using knockoff VIMPs for individual variables can still be challenging to interpret. In such situations we encourage thoughtful consideration of the question of interest, careful variable selection, and examination of meaningful groups of features. For interpretability in classification models, we further encourage computing VIMPs based on sensitivity and specificity instead of relying on accuracy alone.

Our literature review highlights the need for improved VIMP methods in applied medical research, as we found that OOB VIMPs are commonly misused and misinterpreted. The literature review findings also raised serious concerns about replicability. Among the 43 papers that performed analyses with random forests, it would be difficult to identify any two that took the same specific approach in the analysis. In practice, this suggests that two sets of researchers could be given identical datasets and models (e.g., random forests) and yet reach very different conclusions about the role of the features in the models. Further complicating matters is the fact that authors often fail to report what kind of importance metrics are being used and/or how they are calculated. Finally, even if one wanted to attempt to replicate the results, the data were often not publicly available.

Using data from the publicly available Sleep Heart Health Study (SHHS), we examined the contribution of PSG derived features for predicting stroke in a random forest relative to other established and emerging risk factors. Comparing the findings of OOB VIMPs versus knockoff VIMPs serves to highlight important methodological take-home messages. Notably, although there were some consistent features identified across approaches (e.g., age), the overall take-home messages from the two approaches are different. In addition to age, the OOB VIMPs suggested that lung function and height are major risk factors for stroke. This finding is novel, but hard to explain physiologically. On the other hand, the knockoff VIMPs confirmed many variables of known importance and indicated some novel ones that are plausible (the PSG variables). Although a result that is more intuitive does not necessarily imply that it is more correct, the differing findings – combined with the known biases of OOB VIMPs – suggests that implementing modern knockoff VIMP approaches could meaningfully improve the rigor and reproducibility of random forest VIMP findings.

Another important take-home message is highlighted by observing that the top two OOB VIMPs (FEV and FVC) had the second highest pairwise correlation among all pairs of features in the dataset (*r* = 0.94); however, neither feature stood out among the knockoff VIMPs. Bias towards correlated measures is a key limitation of OOB VIMPs. We also note that OOB VIMPs for a classification model implicitly consider a 0.50 probability threshold for stroke prediction, which may not be optimal for an imbalanced outcome such as stroke. In our cross-validation we identified a 0.033 prediction threshold as optimal. Similarly, considering only accuracy in the OOB VIMP is also likely to be misleading. We demonstrated knockoff VIMPs based on more meaningful sensitivity and specificity metrics. Finally, as noted previously, the OOB VIMPs have limited interpretability because they never actually consider the model without each of the listed variables. Thus, they cannot provide information about what would have happened if these variables had not been included in the model.

Strengths of our work include our comprehensive literature review highlighting the extensive use of OOB VIMPs in applied medical research, explanation and recommendation of unbiased and interpretable knockoff VIMP approaches, demonstration of innovative analytic strategies using knockoff VIMPs, and illustration of the notably different findings that may be observed with OOB versus knockoff VIMPs through incident stroke prediction in the SHHS data.

However, these strengths should be considered in the context of some limitations. First, although SHHS had a large sample size, the incidence of stroke was low, which could add to model instability. Whenever cross-validation and resampling is used, we expect findings to differ slightly across iterations. Second, we did not seek to understand the directions of the effects, which inherently dampens clinical interpretability of our findings. Third, our findings should be considered inherently associative and non-causal. They are conditional on the other variables included in the model and the use of the random forest (e.g., versus a linear model). The inclusion of other types of features, or use of a different model, could produce different findings. Last, a non-trivial barrier to widespread use of knockoff VIMPs is computational cost. For the SHHS sample, it required an average of 52 min to calculate a knockoff VIMP for a single variable or domain. With a total of 111 knockoff VIMPs (7 Level 1; 26 Level 2; 78 Level 3), this would require 4 days of continuous processing on a single CPU. To enhance efficiency, we parallelized these tests through the use of servers within the University’s Center for Research Computing, which allowed us to compute over 100 VIMPs simultaneously.

These limitations speak to future directions of our work. It will be important to examine the direction of associations of the novel PSG predictors of stroke. Some approaches to do this within the random forest framework already exist, including plotting marginal effects over the range of the outcome [6]. Methodological efforts should also be made to quantify potential sources of instability from VIMPs and examine the potential impact of missing data imputation on knockoff VIMPs. In our application, there was relatively a small percentage of missing data (median [Q1, Q3] = 0.2% [0%, 6.5%]). However, it is possible that VIMPs of features with larger percentages of missing data may be impacted. Finally, to facilitate the uptake of knockoff VIMP approaches in applied research, it will be essential to develop ways to improve the computational efficiency of knockoff VIMPs, thereby making them a more viable option in comparison to OOB VIMPs, which require dramatically less computational time.

## Supplementary Information

**Additional file 1.**

## Data Availability

The dataset supporting the conclusions of this article is available in the National Sleep Resource repository: [31, 32]. Sleep Heart Health Study—Sleep Data—National Sleep Research Resource—NSRR.

## References

[1] Fernández-Delgado M, Cernadas E, Barro S, Amorim D (2014). Do we need hundreds of classifiers to solve real world classification problems?. J Mach Learn Res.

[2] Breiman L (2001). Random forests. Mach Learn.

[3] Nicodemus KK, Malley JD, Strobl C, Ziegler A. The behaviour of random forest permutation-based variable importance measures under predictor correlation. BMC Bioinformatics. 2010;11:110. 10.1186/1471-2105-11-110. 10.1186/1471-2105-11-110 PMC2848005 20187966

[4] Strobl C, Boulesteix AL, Zeileis A, Hothorn T. Bias in random forest variable importance measures: illustrations, sources and a solution. BMC Bioinformatics. 2007;8:25. 10.1186/1471-2105-8-25. 10.1186/1471-2105-8-25 PMC1796903 17254353

[5] Tolosi L, Lengauer T (2011). Classification with correlated features: unreliability of feature ranking and solutions. Bioinformatics (Oxford, England).

[6] Hooker G, Mentch L, Zhou S (2021). Unrestricted permutation forces extrapolation: variable importance requires at least one more model, or there is no free variable importance. Stat Comput.

[7] Coleman T, Peng W, Mentch L (2022). Scalable and Efficient Hypothesis Testing with Random Forests. J Mach Learn Res.

[8] Williamson BD, Gilbert PB, Simon NR, Carone M. A general framework for inference on algorithm-agnostic variable importance. J Am Stat Assoc. 2021. Epub Ahead of Print. 10.1080/01621459.2021.2003200 PMC10652709 37982008

[9] Tibshirani R (1996). Regression shrinkage and selection via the lasso. J R Stat Soc Ser B.

[10] Quan SF, Howard BV, Iber C, et al. The Sleep Heart Health Study: design, rationale, and methods. Sleep. 1997;20(12):1077–1085. Not in File. 9493915

[11] Yaggi HK, Concato J, Kernan WN, Lichtman JH, Brass LM, Mohsenin V (2005). Obstructive sleep apnea as a risk factor for stroke and death. N Engl J Med.

[12] Culebras A, Anwar S. Sleep Apnea Is a Risk Factor for Stroke and Vascular Dementia. Curr Neurol Neurosci Rep. 2018;18(8):53. 10.1007/s11910-018-0855-1. 10.1007/s11910-018-0855-1 29938308

[13] McDermott M, Brown DL (2020). Sleep apnea and stroke. Curr Opin Neurol.

[14] Redline S, Yenokyan G, Gottlieb DJ (2010). Obstructive sleep apnea-hypopnea and incident stroke: the sleep heart health study. Am J Respir Crit Care Med.

[15] Gottlieb E, Landau E, Baxter H, Werden E, Howard ME, Brodtmann A (2019). The bidirectional impact of sleep and circadian rhythm dysfunction in human ischaemic stroke: A systematic review. Sleep Med Rev.

[16] McDermott M, Brown DL, Chervin RD (2018). Sleep disorders and the risk of stroke. Expert Rev Neurother.

[17] Qi W, Ma J, Guan T, et al. Risk Factors for Incident Stroke and Its Subtypes in China: A Prospective Study. J Am Heart Assoc. 2020;9(21):e016352. 10.1161/jaha.120.016352. 10.1161/JAHA.120.016352 PMC7763402 33103569

[18] O'Donnell MJ, Chin SL, Rangarajan S (2016). Global and regional effects of potentially modifiable risk factors associated with acute stroke in 32 countries (INTERSTROKE): a case-control study. Lancet.

[19] Alloubani A, Saleh A, Abdelhafiz I (2018). Hypertension and diabetes mellitus as a predictive risk factors for stroke. Diabetes Metab Syndr.

[20] Guzik A, Bushnell C. Stroke Epidemiology and Risk Factor Management. Continuum (Minneap Minn). 2017;23(1, Cerebrovascular Disease):15–39. 10.1212/con.0000000000000416. 10.1212/CON.0000000000000416 28157742

[21] Sarikaya H, Ferro J, Arnold M (2015). Stroke prevention–medical and lifestyle measures. Eur Neurol.

[22] Breiman L (1996). Bagging Predictors. Mach Learn.

[23] Kursa MBaJ, A. and Rudnicki, W. Boruta - A System for Feature Selection. Fundamenta Informaticae. 2010;101:271-285.

[24] Wallace ML, Coleman TS, Mentch LK, et al. Physiological sleep measures predict time to 15-year mortality in community adults: Application of a novel machine learning framework. J Sleep Res. 2021:e13386. 10.1111/jsr.13386. 10.1111/jsr.13386 PMC8591145 33991144

[25] Candes E, Fan Y, Janson L, Lv J (2018). Panning for gold:‘model-X’knockoffs for high dimensional controlled variable selection. J R Stat Soc Ser B (Statistical Methodology).

[26] Patterson E, Sesia M. knockoff: The knockoff filter for controlled variable selection. R package version 0.3.6. 2022. https://CRAN.R-project.org/package=knockoff.

[27] Mentch LaZ S (2022). Getting better from worse: Augmented bagging and a cautionary tale of variable importance. J Mach Learn Res.

[28] Wasserstein RL, Lazar NA (2016). The ASA's statement on p-values: context, process, and purpose. Am Stat.

[29] Stekhoven DJ, Bühlmann P (2012). MissForest–non-parametric missing value imputation for mixed-type data. Bioinformatics (Oxford, England).

[30] Wright MNaZ, A. ranger: A fast implementation of random forests for high dimensional data in C++ and R. J Stat Softw. 2017;77:1-17.

[31] Zhang GQ, Cui L, Mueller R (2018). The National Sleep Research Resource: towards a sleep data commons. J Am Med Inform Assoc.

[32] Dean DA, Goldberger AL, Mueller R (2016). Scaling Up Scientific Discovery in Sleep Medicine: The National Sleep Research Resource. Sleep.

