# Supplementary material for: Use and misuse of random forest variable importance metrics in medicine: demonstrations through incident stroke prediction
Source: BMC Med Res Methodol. 2023 Jun 19;23:144. doi: 10.1186/s12874-023-01965-x (PMC10280951; doi:10.1186/s12874-023-01965-x)
Supplement: Supplementary file 1 — Additional file 1. [file 12874_2023_1965_MOESM1_ESM.docx]

**Supplemental Materials**

Use and Misuse of Random Forest Variable Importance Metrics in Medicine: Demonstrations using Incident Stroke Prediction

Meredith L. Wallace, Lucas Mentch, Bradley J. Wheeler, Amanda A. Tapia, Marc Richards, Siyu Zhou, Lixia Yi, Susan Redline, Daniel J Buysse

S1. Constructing a Random Forest

Here we provide a non-technical summary of random forest methods. The basis of a random forest is a tree model, which is developed by *empirically* identifying the feature and associated binary cut-off point that optimally divides the sample into two subgroups with different outcomes (e.g., higher vs. lower risk of incident stroke). This data-driven splitting procedure continues iteratively on each successive subgroup until a pre-defined stopping rule is met (e.g., a minimum subgroup sample size or maximum “depth” of tree). Each final subgroup in the tree is associated with an estimated outcome (e.g., predicted risk of stroke).

To demonstrate, we fit a single tree model on Sleep Heart Health Study (SHHS) data to predict incident 5-year stroke using the rpart function and package in R^3^ (**Figure S1**). Participants who are older age (≥72), have more role limitations due to emotional problems (Short Form 36 Health Survey Questionnaire [SF-36]^4,5^ Role Limitations due to Emotional Problems < 54), and reduction in airflow during sleep due to apneic or hypopneic events as measured by PSG (Apnea-Hypopnea Index ≥ 1.1) had the highest stroke risk (24% of N=131 SHHS participants).

Although single tree models can be highly interpretable when relatively few splits are made, they are rarely optimal in terms of predictive accuracy and the specific structure of the tree tends to be highly variable in the sense that small changes in the data can are rarely optimal in terms of predictive accuracy and lead to substantially different^1,6^. Random forests address these limitations by resampling the participants in original data hundreds of times and growing an individual tree model on each sample. The results from each tree are aggregated – averaged in the case of a continuous outcome – to produce a final prediction (e.g., see **Figure S2**).^1^ Importantly, trees in a random forest incorporate additional randomness by selecting only a subset of the variables to consider for each split (the optimal number/proportion of features to consider can be set to a default value or, ideally, selected via cross-validation). This randomness arising through both the data resampling and the random feature selection serves to reduce correlations among the individual models, thereby reducing the variance in the final averaged estimate. This very often produces more accurate predictions than could be obtained from a single tree model, especially when the data are very noisy^2^. For further details on trees and random forests, we refer the reader to the original publication.^1^

**Figure S1.** Tree-structured model for stroke prediction in SHHS.

**
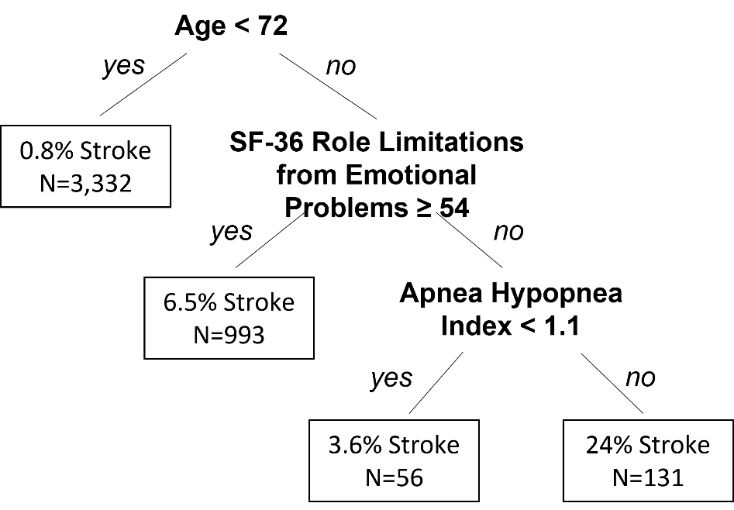
**

**Figure S2.** Illustration of a random forest as a collection of trees.


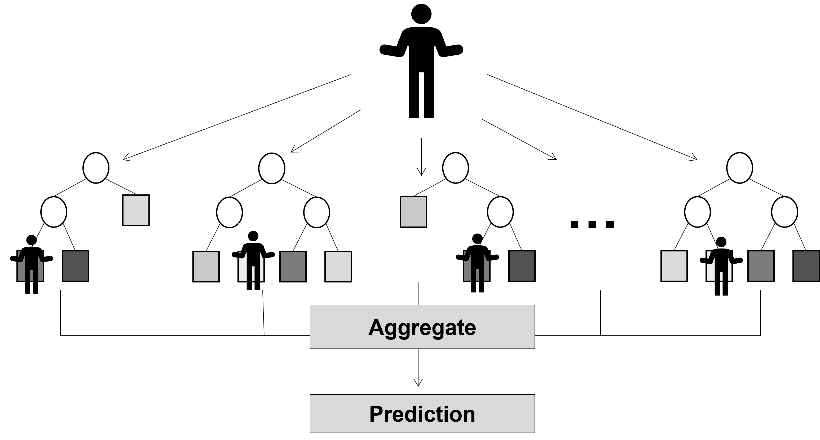


S2. Full findings from the SHHS Demonstration

**Figure S3** provides full results from the SHHS demonstration for stroke prediction.

**Figure S3**. Sensitivity and Specificity VIMPs for all features and domains.


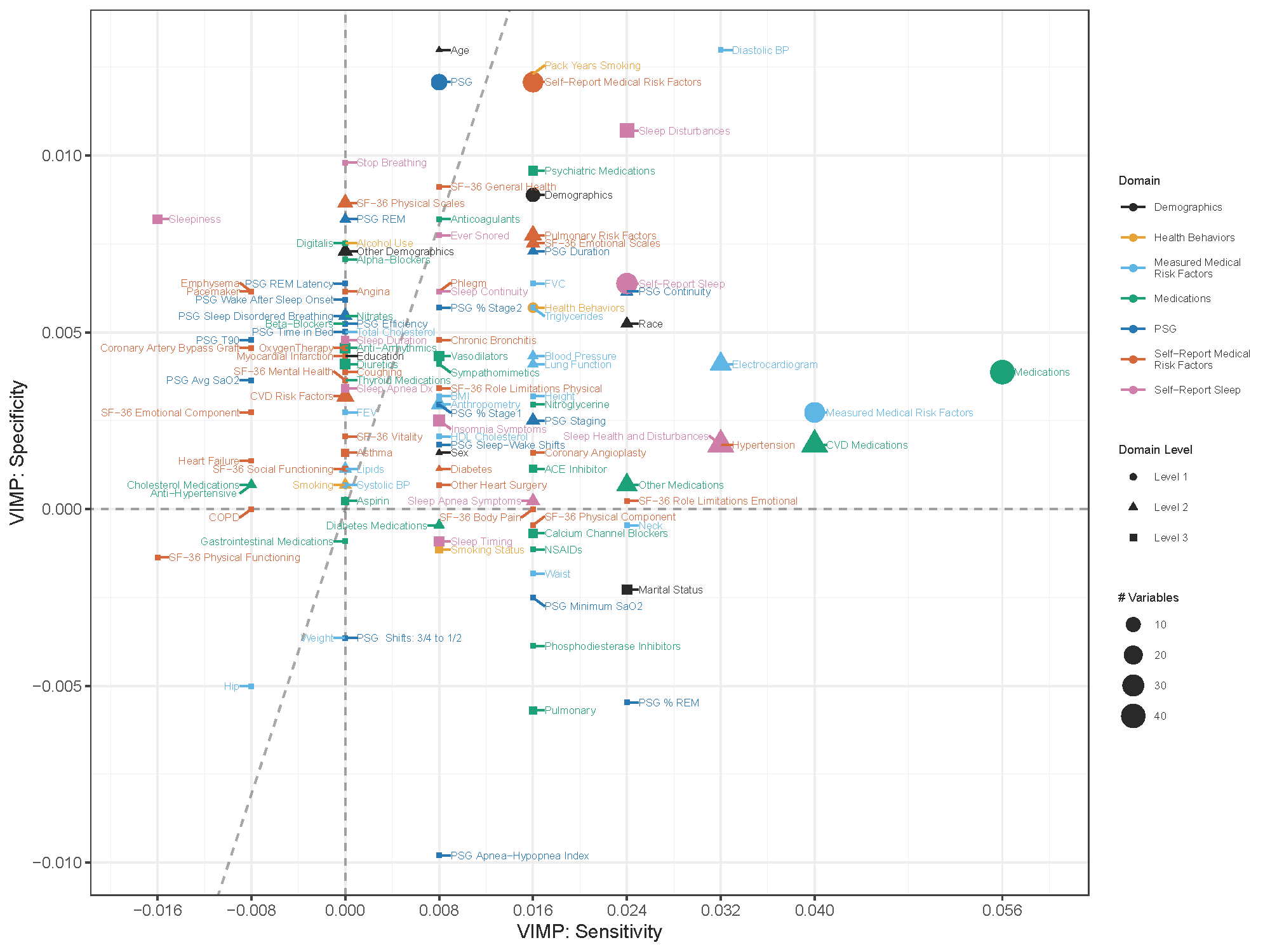


**References**

1. Breiman L. Random forests. *Machine Learning*. 2001 2001;45:5-32.

2. Mentch L, Zhou S. Randomization as Regularization: A Degrees-of-Freedom Explanation for Random Forest Success. *Journal of Machine Learning Research*. 2021;21(171):1-36.

3. *rpart: Recursive Partitioning and Regression Trees*. 2022. <https://CRAN.R-project.org/package=rpart>

4. Ware J, Kosinski M, Gandek B. *SF-36 Health Survey: Manual and Interpretation Guide*. 2000.

5. Ware JE, Jr., Sherbourne CD. The MOS 36-item short-form health survey (SF-36). I. Conceptual framework and item selection. *Medical care*. Jun 1992;30(6):473-83.

6. Hastie T, Tibshirani R, Friedman J. *The Elements of Statistical Learning: Data Mining, Inference, and Prediction*. 2 ed. Springer-Verlag; 2009.
